# Carbon monoxide neurotoxicity is triggered by oxidative stress induced by ROS production from three distinct cellular sources

**DOI:** 10.1016/j.redox.2022.102598

**Published:** 2023-01-07

**Authors:** Plamena R. Angelova, Isabella Myers, Andrey Y. Abramov

**Affiliations:** Department of Clinical and Movement Neurosciences, UCL Queen Square Institute of Neurology, Queen Square, London, WC1N 3BG, UK

**Keywords:** CO toxicity, Brain, ROS, NADPH oxidase, Neuron, Astrocyte, DNS, HBOT, CORM-401, Mn(CO)_4_{S_2_CNMe(CH2CO_2_H), NADPH, oxidase**-**reduced nicotinamide adenine dinucleotide phosphate oxidase, AEBSF, 4-(2 aminoethyl) benzenesulfonyl fluoride hydrochloride, DPI, Diphenyleneiodonium, GSH, glutathione, MCB, monochlorobimane, FCCP, carbonyl cyanide 4 (trifluoromethoxy)phenylhydrazone

## Abstract

Carbon monoxide (CO) poisoning is one of the leading causes of toxic mortality and morbidity. We have studied the generation of reactive oxygen species in cortical neurons in culture in response to toxic doses of CO exposure. Fluorescence microscopy was used to measure the rate of free radical generation, lipid peroxidation, GSH level and also mitochondrial metabolism. We have found that toxic concentrations of CO released from CORM-401 induced mitochondrial depolarisation and inhibition of NADH dependent respiration to a lesser degree than when compared to ischaemia. Energy collapse was not observed within 40 min of CO exposure. We have found that CO induces the generation of reactive oxygen species resulting in lipid peroxidation and a decrease in GSH via three different mechanisms: from mitochondria during the first minutes of CO exposure, from xanthine oxidase at around 20 min exposure due to energy deprivation, and considerable ROS production from NADPH oxidase in the post CO exposure period (re-oxygenation). Inhibition of these different phases with mitochondrial antioxidants, inhibitors of xanthine oxidase, or NADPH oxidase, protected neurons and astrocytes against CO-induced oxidative stress and cell death. The most profound effect was seen during NADPH oxidase inhibition. Thus, oxidative stress has a remarkably significant role in CO-induced neuronal cell death and preventing its occurrence during reoxygenation is of great importance in the consideration of a positive, neurologically protective therapeutic outcome for CO exposed patients.

## Highlights

• Carbon monoxide induce production of ROS from different cellular sources: mitochondria, xanthine oxidase and NADPH oxidase.
• CO-induced ROS production stimulate oxidative stress with maximal implication of NADPH oxidase.
• CO-induced oxidative stress in neurons and astrocytes is a trigger for neuronal cell death.

## Graphical abstract

**Figure:**
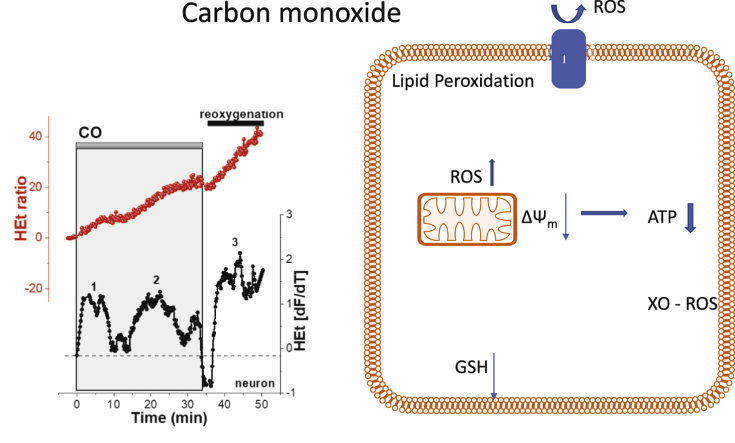

## 1 Introduction

Carbon monoxide (CO) poisoning is a common cause of morbidity and mortality worldwide. Whilst it is one of the most common lethal poisonings, neurological or psychiatric sequelae occurs in up to 67% of survivors. This suggests that CO is implicated in neuronal death, or loss of function of the cells in the central nervous system. Approximately half of those who survive severe CO poisoning develop delayed neurological sequelae after a latency period of 2–40 days, with varied clinical manifestations, persistent neuropsychological effects, and no guarantee of complete recovery. Such effects have also been recorded in patients who experience more moderate levels of poisoning, with changes not always evident on MRI. Therefore, prompt and adequate treatment following poisoning is essential, with a focus on neuroprotection required. CO affects the oxygen carrying capacity of the blood as it binds with haemoglobin to form carboxyhaemoglobin, so preventing the uptake of oxygen and the release of any available oxygen to the tissues. CO produces effects similar to chemical hypoxia, when oxygen-dependent processes are blocked despite there being an abundance of oxygen, causing severe hypoxia in tissues. As less oxygen is available to the tissues, cardiac, neuropsychological, and other tissue functions are negatively affected. CO has been also shown to inhibit mitochondrial respiration, a major consumer of oxygen in the cells. These conditions cause tissue toxicity and in cases of CO poisoning, make the reintroduction of oxygen essential for recovery. In considering this point, treatment with 100% hyperbaric oxygen (HBOT) is recommended (in countries other than the UK), because it reduces carboxyhaemoglobin dissociation half-life from more than 4 h in room air (or 45 min on 100% oxygen) to 23 min at 2.5 atm absolute (ATA). However, post CO poisoning, re-oxygenation by 100% HBOT or 100% normobaric oxygen, induces neurotoxicity [14,30]. This type of neurotoxicity can be explained, in part, by the same mechanisms of neuronal cell death which are induced by anoxic or hypoxic conditions.

Episodes of hypoxia/anoxia are shown to be one of the major triggers for neuronal cell death in various neurological diseases. Several mechanisms which prompt neuronal cell death under these conditions were suggested, e.g. energy collapse due to lack of ATP production, glutamate excitotoxicity and oxidative stress [29,33,34]. Oxidative damage is induced by the overproduction of reactive oxygen species (ROS) and/or by the reduction of the antioxidant defence in the cell [15]. Overproduction of ROS in ischaemia/reperfusion and oxidative stress is orchestrated by multiple enzymatic ROS sources that all have an impact on ischaemia-induced cell death A. Y [3]. However, production of ROS is not only a pathological event but also a trigger of physiological redox signal [5,6,9]. Moreover, endogenous CO is also shown to play a physiological role in central nervous and cardiovascular systems [19]. Massive changes in ROS production and levels of CO may possibly alter the signalling pathways of ROS and CO in neurons that also can contribute to the development of neurological or neuropsychiatric pathology.

Mitochondria are the major targets for CO- and ischaemia-induced neuronal damage. It has been shown that CO directly inhibits cytochrome C in mitochondria, and similarly inhibits cytochrome C through a lack of oxygen in an anoxia/hypoxia setting [4,11,21,36]. Inhibition of mitochondrial respiration not only blocks ATP production in oxidative phosphorylation, but can also induce ROS production as a result of electron leak from the electron transport chain of mitochondria [1,20].

The elevated level of neuronal toxicity at the time of oxygen re-introduction after CO poisoning is suggestive of the importance that should be attributed to oxidative damage in CO-induced neurotoxicity. In considering this, we have studied the effect of CO on mitochondrial metabolism, the major consumer of oxygen, and the role of oxidative stress in the mechanism of CO-induced neuronal cell death.

## 2 Materials and methods

### 2.1 Primary neuronal culture preparation

Mixed neuronal brain cultures were prepared from Sprague-Dawley rat pups 0–3 days postpartum (UCL breeding colony). Animal husbandry and experimental procedures were performed in full compliance with the United Kingdom Animal (Scientific Procedures) Act of 1986. Subjects were culled using a Schedule 1 procedure and the brain was dissected into ice-cold HEPES buffered salt solution (Ca^2+^, Mg^2+^-free; Gibco-Invitrogen, Paisley, UK). The tissue was minced and trypsinized (0.25% for 15 min at 37 °C), triturated and plated on poly-d-lysine-coated 22 mm coverslips and cultured in Neurobasal-A medium (Gibco-Invitrogen) supplemented with B-27 (Gibco-Invitrogen) and 2 M Glutamax using routine protocol [28]. Cultures were maintained at 37 °C in a humidified atmosphere of 5% CO_2_ and 95% air, fed once a week and maintained for a minimum of 14 days before experimental use. Neurons were easily distinguishable from glia: they appeared bright using phase contrast, had smooth rounded somata and distinct processes, and lay just above the focal plane of the glial layer. Cells were used at 14–16 days *in vitro*.

### 2.2 CO

CORM-401, a Mn-containing water-soluble CO-releasing molecule releasing at least 3 mol of CO molecules per 1 mol of the compound (half-life of 13–14 min), was used to deliver CO [22,23]. Inactive CORM-401 (iCORM-401; contains MnSO_4_ and the ligand for CORM-401), which does not liberate CO, was used to exclude the effects of components of the moiety other than CO .

### 2.3 Imaging of mitochondrial membrane potential

Cortical neurons were loaded for 20 min at room temperature with Rhodamine123 (1 μM, Molecular Probes) in HEPES-buffered salt solution (HBSS) composed (mM): 156 NaCl, 3 KCl, 2MgSO_4_, 1.25 KH_2_PO_4_, 2 CaCl_2_, 10 glucose and 10 HEPES, pH adjusted to 7.35 with NaOH, and the cells were then washed 3–5 times before experiment.

Fluorescence measurements were obtained on an epifluorescence inverted microscope equipped with a 20× fluorite objectives. Δψ_m_ was monitored in single cells using excitation light provided by a Xenon arc lamp, the beam passing sequentially through 10 nm band pass filters centred at 490 nm housed in computer-controlled filter wheel (Cairn Research, Kent, UK). Emitted fluorescence light was reflected through a 515 nm long-pass filter to a cooled CCD camera (Retiga, QImaging, Canada). All imaging data were collected and analysed using software from Andor (Belfast, UK). Accumulation of Rh123 in polarised mitochondria quenches the fluorescent signal in cytosol; in response to depolarisation the fluorescence signal is de-quenched; an increase in Rh123 signal in the whole neuron or astrocyte therefore indicates mitochondrial depolarisation. We normalised the signals between resting level (set to 0) and a maximal signal generated in response to the protonophore FCCP (1 μM; set to 100%).

### 2.4 MagFura-2 measurements

To assess the ATP levels, which correlate with Mg^2+^ changes, [Mg^2+^] was imaged using MagFura-2 AM. Fluorescence images were acquired (10 s interval) on an epifluorescence inverted microscope equipped with a 20× fluorite objective (excitation at 340 and 380 nm). The emitted light was reflected through a 515 nm long-pass filter to a cooled CCD camera (Retiga; QImaging) and digitised to 12-bit resolution (Cairn Research, UK). Andor iQ3 was employed for data collection and analysis.

### 2.5 NADH measurements

NADH autofluorescence was measured using an epifluorescence inverted microscope equipped with a × 40 fluorite objective. Excitation light at a wavelength of 360 nm was provided by a Xenon arc lamp, the beam passing through a monochromator (Cairn Research, Faversham, Kent, UK). Emitted fluorescence light was reflected through a 455 nm long-pass filter to a cooled CCD camera (Retiga, QImaging) and digitised to 12 bit resolution. Imaging data were collected and analysed using software from Andor (Belfast, UK).

### 2.6 ROS production assessment

Fluorescence measurements were obtained on an epifluorescence inverted microscope equipped with a 20 × fluorite objective. Excitation at 540 nm and emission recorded above 560 nm were used to quantify the oxidized form (ethidium), whereas excitation at 360 nm and emission collected from 405 nm to 470 nm was used for the reduced form (hydroethidium). For HEt and MitoSOX measurements, ratios of the oxidized to reduced forms of the dye were measured. The traces are presented as ratio 540nm/360 nm. All data reported in this study were obtained from at least five coverslips and 2–3 different cell and sample preparations.

For measurement of mitochondrial ROS production, cells were pre-incubated with MitoSOX (5 μM; Molecular Probes, Grand Island, NY, USA) for 10 min at room temperature. For measurement of cytosolic ROS production, dihydroethidium (HEt, 2 μM) was present in the solution during the experiment. No pre-incubation (“loading”) was used for HEt to limit the intracellular accumulation of oxidized products [7].

### 2.7 GSH level assessment

Co-cultures of neurons and astrocytes were incubated with 50 μM monochlorobimane (MCB) (Molecular Probes, Invitrogen) for 40 mins in HEPES buffered salt solution prior to imaging. Cells were then washed with HEPES buffered salt solution and images of the fluorescence of the MCB-GSH were acquired using a Zeiss UV–vis 710 CLSM with excitation at 405 nm and emission at 435–485 nm.

### 2.8 Lipid peroxidation assay

The rate of lipid peroxidation was measured using confocal microscopy. Confocal images were obtained with a Zeiss 710 LSM with an integrated META detection system. To assess lipid peroxidation C11-BODIPY (581/591, 2 μM, Molecular probes) was excited using the 488 and 543 nm laser line and fluorescence measured using a band-pass filter from 505 to 550 nm and 560 nm long-pass filter (40× objective). Illumination intensity was kept to a minimum (at 0.1–0.2% of laser output) to avoid phototoxicity, and the pinhole set to give an optical slice of ∼2 μm. Addition of a bright field image allowed separation between neurons and glia, that are visibly different and are situated on different focal planes. Data were acquired and analysed using ZEN2009 software.

### 2.9 Statistical analysis

Statistical analysis (unpaired two sample *t*-test, or one-way analysis of variance (ANOVA), p value set at 0.05) and curve fitting were performed using Origin 2021 (Microcal Software Inc., Northampton, MA) software. Results are expressed as means ± standard error of the mean (SEM). N = number of culturing conditions and n = number of cells, if not stated otherwise. Sample sizes for experiments were selected to capture adequate technical variation (number of cells; numbers of fields of view; number of coverslips). All experiments were repeated a minimum of three times.

## 3 Results

### 3.1 CO induces mitochondrial depolarisation and inhibition of mitochondrial respiration in neurons and astrocytes

CO not only prevents oxygen delivery to the tissues by binding to haemoglobin but further binds to cytochrome C causing the inhibition of mitochondrial respiration [4].

**Fig. 1:**
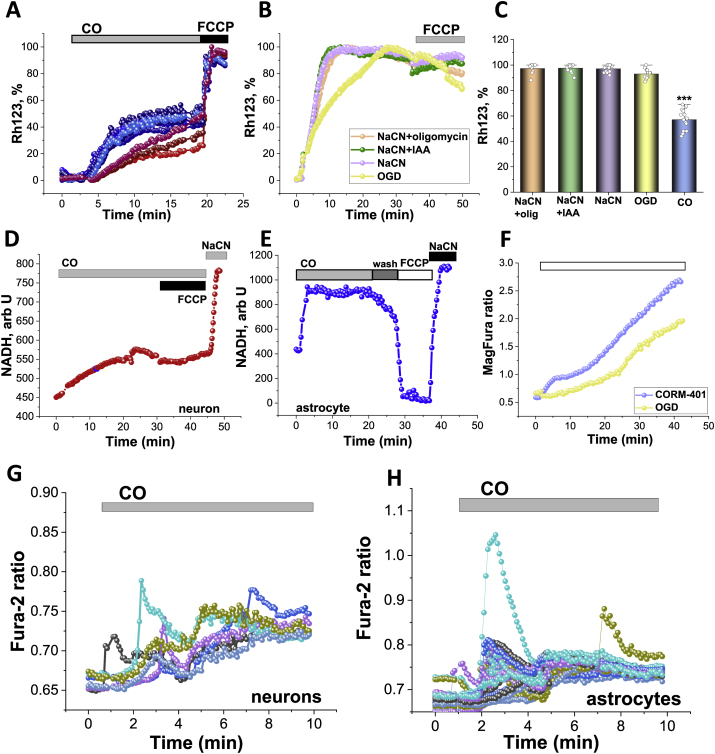
**CO induces inhibition of the mitochondrial electron transport chain, mitochondrial depolarisation and increased ATP consumption. A,** Application of 60 μM CORM-401 induces an increase in Rh123 fluorescence. **B**, Effects of 1 mM NaCN alone and in combination with 20 μM Iodoacetic Acid (IAA) or 2 μg/ml oligomycin and oxygen-glucose deprivation (OGD) on the Rh123 fluorescence of neurons and astrocytes. **C** Quantification bar charts of chemical ischaemia, OGD and 60 μM CORM-401 on the Rh123 fluorescence after 20 min of application (normalised to cellular response to 1 μM FCCP) in primary neurons and astrocytes from co-culture. NADH autofluorescence in single representative neuron (**D**) and astrocyte (**E**) upon CO application. **F,** Mag-Fura-2 ratio in single representative neurons upon application of 60 μM CORM-401 or OGD (blank bar represents CO exposure or OGD). Changes in Fura-2 ratio of several neurons (**G**) and astrocytes (**H**) in response to 60 μM CORM-401. Data are represented as mean ± SEM. ***p < 0.0001.

In our experiments, CO release from CORM-401 (60 μM), but not from iCORM-401 (inactivated CORM-401, 60 μM), induced a fast and progressive increase in Rh123 fluorescence in cortical neurons and astrocytes (n = 89 neurons; n = 101 astrocytes; Fig. 1 A) that corresponds to a loss of mitochondrial membrane potential (Δψm) and was confirmed by the application of the protonophore FCCP at the end of the experiments (Fig. 1 A). Importantly, CO induces significant but incomplete mitochondrial depolarisation in neurons and astrocytes after 20 min of acute exposure (Rh123 signal rose to 56 ± 4%, 100% - complete depolarisation after 1 μM FCCP; N = 8 experiments; Fig. 1 A, C). The effect of CO on the mitochondrial membrane potential of neurons and astrocytes was much lower compared to those evoked by chemical hypoxia – application of 1 mM NaCN +20 μM IAA; or 1 mM NaCN +2 μg/ml oligomycin; or 1 mM NaCN alone - all combinations induced almost complete mitochondrial depolarisation (Fig. 1 B, C; N = 6 experiments). Importantly, oxygen-glucose deprivation (OGD; replacement of oxygen by argon in HBSS lacking glucose) also induced a much higher effect on Δψm of neurons and astrocytes compared to the acute application of CO (N = 3; Fig. 1 B, C). Thus, toxic concentrations of CO dramatically decrease mitochondrial membrane potential in both neurons and astrocytes, but it does not induce a complete loss of Δψm as found with chemical ischaemia or OGD.

Further, the effect of acute CO application on the mitochondrial respiration of live primary neurons and astrocytes was assessed using measurement of the NADH autofluorescence [10]. NADH is produced in the Krebs cycle in the matrix of mitochondria, and is used as a substrate and a donor of electrons for complex I of the electron transport chain [1]. In our experiments, application of 60 μM CORM-401 induced a slow and progressive increase in NADH fluorescence in neurons (n = 116 neurons; Fig. 1D). Addition of mitochondrial uncoupler FCCP (1 μM) to these cells in the presence of CO did not induce further activation of mitochondrial respiration and a decrease in NADH fluorescence confirmed full impairment of mitochondrial respiration by CO (Fig. 1D). Consequent inhibition of mitochondrial respiration with 1 mM NaCN at the end of the experiment induced a rise in NADH autofluorescence signal (Fig. 1D). However, 1 μM FCCP induced activation of mitochondrial respiration and a decrease in NADH autofluorescence when this uncoupler was applied to the cells after washing the CORM-401 out of the medium (Fig. 1 E). It should be noted that the effect of CO on mitochondrial NADH in astrocytes from co-culture was faster and larger in amplitude compared to the effect of CO in neurons (n = 143; Fig. 1D and E). These results strongly suggest that CO induces extensive, but not complete inhibition of mitochondrial respiration in neurons and astrocytes from primary co-culture, and their respiration could be recovered after removal of CO from the medium.

### 3.2 CO induces consumption of ATP but does not lead to cell lysis due to energy deprivation

ATP is stored as a magnesium complex in the cells. Upon hydrolysis of ATP, Mg^2+^ is released from the MgATP complex, and therefore measurement of the changes in the cellular free magnesium using the Mg^2+^ sensitive fluorescent probe MagFura-2, can be used as an indirect indicator of the ATP consumption [26,38]. Application of inhibitors of glycolysis and/or oxidative phosphorylation blocks ATP production in cells, which leads to utilisation of the available ATP in the cell, and a subsequent Mg^2+^ release. In addition to binding to Mg^2+^, the MagFura-2 dye is also a low-affinity Ca^2+^ indicator that can help detect high cytosolic calcium rise at the time of cell lysis, i.e. the energetic collapse due to a total cellular ATP depletion and the inability of the cell to maintain Ca^2+^ homeostasis, enables the estimation of the cellular energy capacity. In our experiments, application of 60 μM CORM-401 produced a slowly progressive increase in the MagFura-2 ratio in neurons and astrocytes (N = 4; Fig. 1F) with a secondary increase after 20 min of CO exposure. Interestingly, the effect of OGD on ATP consumption was smaller, and the secondary increase in MagFura-2 ratio occurred approximately 10 min later compared to the initial effect of CO (N = 3; Fig. 1F). It should be noted that exposure of neurons and astrocytes to OGD or CO did not induce cell lysis and consequent cell death from energy deprivation; this results in a peak-like increase in the MagFura-2 ratio, *see* Ref. [27]. Thus, CO activates the consumption of the available ATP, but does not lead to an energy collapse within 40 min of observation.

Ischaemic conditions have been known to induce calcium signal in neurons and astrocytes due to a release of glutamate and ATP [3,8]. Physiological concentration of CO has also been known to induce calcium signal in various cells [23]. Application of toxic doses of CORM-401 (60 μM), but not of iCORM-401, induced calcium signal in neurons (n = 46; Fig. 1G) and in neighbouring astrocytes (n = 39; Fig. 1H).

### 3.3 Carbon monoxide evokes multiphasic increase in ROS production in neurons and in astrocytes

**Fig. 2:**
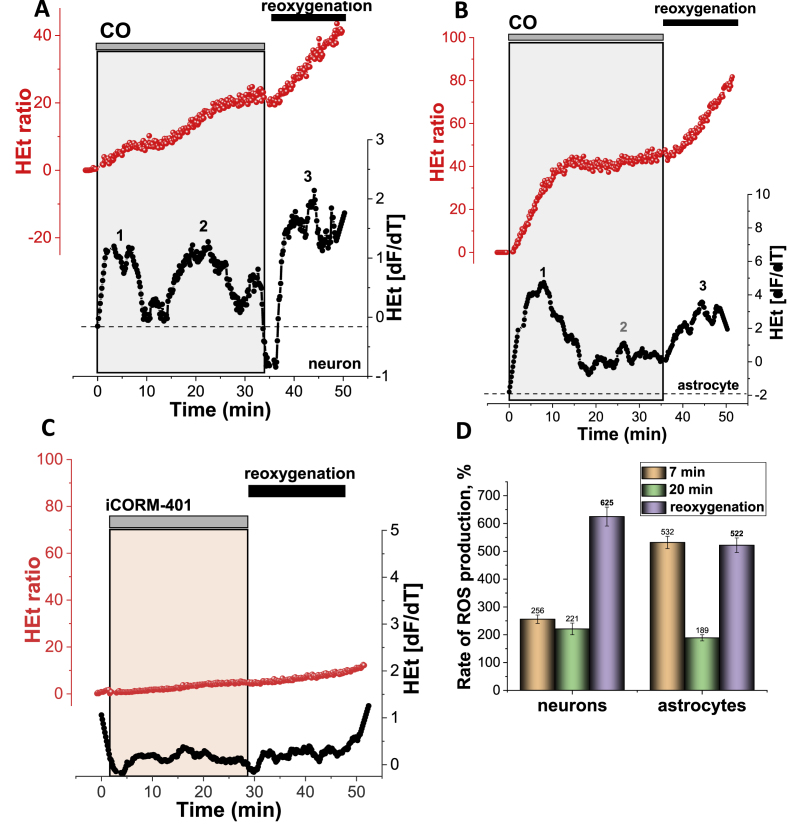
**Carbon monoxide induces distinctive multiphasic activation of ROS production in primary cortical neurons and astrocytes.** Representative traces (**A**-**C,** in red) of fluorescence measurements from single representative cortical neurons (**A**) or astrocyte (**B**) in the continual presence of hydroethidine (HEt, 2 μM). The lower traces (in black) in all panels depict the HEt signal after differentiation. The key phases of ROS generation (or the time at which they would be expected in control experiments) are indicated as ‘**1**’,‘**2**′, ‘**3**’. **C**, Effect of 60 μM iCORM-401 (inactivated CORM-401) on the ROS production in a cortical neuron (representative trace from a single cell). The histogram shown in **D** summarises the data from the mean rates of ROS production at different times of CO exposure (orange 7min, green 20 min and lilac-after reoxygenation) of cortical neurons and astrocytes. Data shown as percentage of the basal rate of HEt fluorescence in control cells (100%). Data are represented as mean ± SEM. (For interpretation of the references to colour in this figure legend, the reader is referred to the Web version of this article.)

Acute exposure of primary co-culture of neurons and astrocytes to toxic doses of CO and subsequent reoxygenation, induced a multiphasic increase in ROS production that was different for neurons and astrocytes. In astrocytes, CO induces an almost immediate 5.32-fold increase in ROS production within the first 7–8 min, followed by a reduction of the rate of the DHE fluorescence to almost the basal rate, with a further activation of ROS production after 20–25 min of CO exposure (N = 10 experiments; Fig. 2B,D). Importantly, washing the CORM-401 out of the medium (reoxygenation) induced a further 5-fold increase in cytosolic ROS production compared to the basal rate (Fig. 2 B, D).

In neurons, activation of ROS production within the first 5 min after CO application was also significant, but less than in the neighbouring astrocytes (256% of the basal rate; N = 10 experiments; Fig. 2A). This initial increase was also followed by partial recovery to the basal rate for 10 min, followed by a profound acceleration in ROS generation (Fig. 2A, D). Activation of the ROS production in neurons was dramatic, where a 6.2-fold increase was seen after washing the CORM-401 out of the medium (Fig. 2 A, D). It should be noted that the inactivated form of CORM (iCORM-401) had no effect on the ROS production in either neurons or astrocytes (N = 4; Fig. 2C). Thus, exposure of primary neurons and astrocytes to CO induced a multiphasic activation of ROS production.

### 3.4 CO-induced ROS production in mitochondria within the first 10 min of CO exposure

**Fig. 3:**
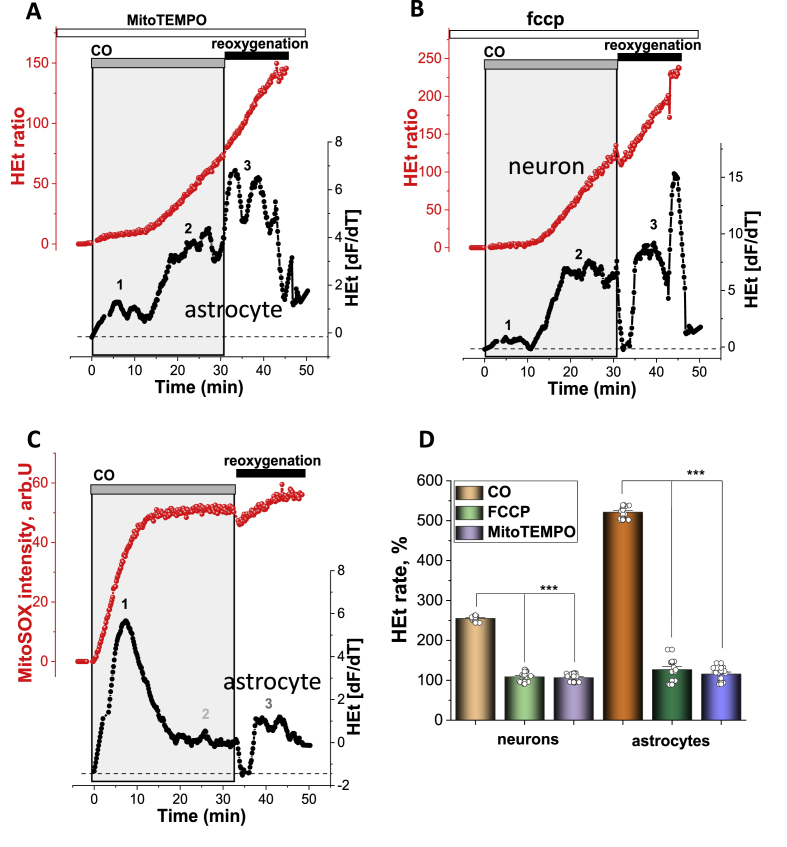
**Mitochondria produce ROS in neurons and astrocytes in the first minutes of CO exposure. A**, Effect of pre-incubation (20 min) of astrocytes with 100 nM MitoTEMPO on CO-induced ROS production (representative trace from single astrocyte, red). **B**, Effect of the pre-incubation of the cells with 0.5 μM FCCP on CO-induced changes in the rate of HEt fluorescence (representative trace from a single neuron, red). **C**, 60 μM CORM-401 induced activation in the rate of MitoSOX fluorescence (representative trace from a single astrocyte, red). The lower traces in all panels (A-C, in black) depict the HEt or MitoSOX signal after differentiation. The histogram shown in **D** summarises the data showing the mean rates of ROS production in the first 10 min of CO exposure in cortical neurons and astrocytes in control and after pre-incubation with 0.5 μM FCCP or 100 nM MitoTEMPO, shown as percentage from a basal rate of HEt fluorescence in control cells (100%). Data are represented as mean ± SEM. ***p < 0.0001. (For interpretation of the references to colour in this figure legend, the reader is referred to the Web version of this article.)

Inhibition of mitochondrial respiration leads to ROS production in mitochondria [6]. Previously, we have demonstrated that the first minutes of ischaemia are characterised by a decrease in Δψm and ROS production in mitochondria [9]. In order to investigate the impact of CO on ROS production from mitochondria, we pre-incubated cells with mitochondrial antioxidant MitoTEMPO (20min, 20 μM). Mitochondrial antioxidant significantly delayed the CO-induced ROS production (Fig. 3 A) and completely blocked the first phase of ROS production. However, incubation of the neurons and astrocytes with MitoTEMPO did not protect cells against the secondary activation of ROS production and the acceleration of ROS production after washing CORM-401 out of the medium (Fig. 3A). Mild uncoupling of mitochondria with low concentration of protonophores has also been shown to decrease mitochondrial ROS production [3,12]. Pre-incubation (10 min) of neurons and astrocytes with 0.5 μM FCCP had an effect similar to that of MitoTEMPO on CO-induced ROS production; it reduced the ROS production only within first the 10 min of CORM-401 applications (N = 4 experiments; Fig. 3B).

To assess the effect of CO on the ROS production in the matrix of mitochondria, we used the fluorescent indicator MitoSOX [7]. As with the results obtained using the fluorescent probe HEt, mitochondrial antioxidant, and uncoupler FCCP, the exposure of neurons and astrocytes to CO induced an increase in mitochondrial ROS production in the first 7–10 min, and a small increase at the time of washing CORM-401 out of solution (Fig. 3C). Thus, mitochondria produce ROS within the first few minutes of an application of toxic doses of CO.

### 3.5 The second phase of CO-mediated activation of ROS production in neurons and astrocytes depends on xanthine oxidase

**Fig. 4:**
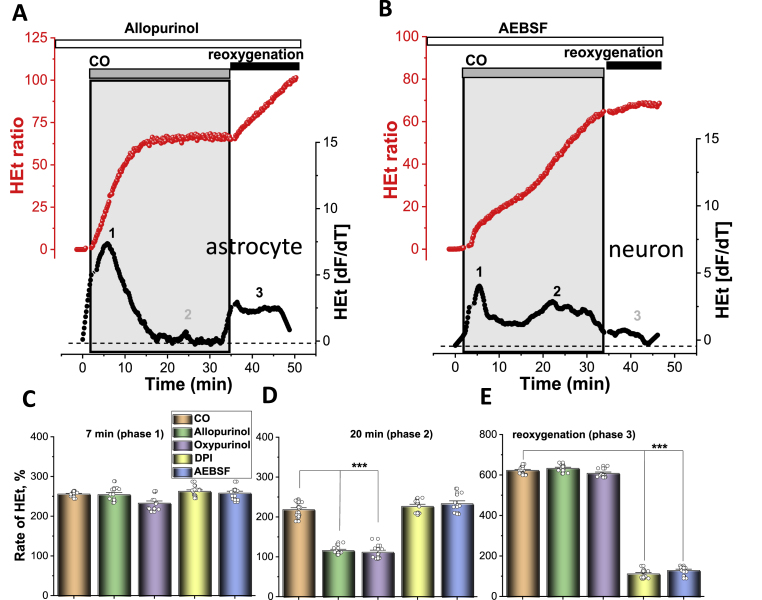
**Cytosolic ROS production in neurons and astrocytes during the CO exposure. A**, Effect of pre-incubation (20 min) of astrocytes with Allopurinol (20 μM) on CO-induced ROS production (representative trace from single astrocyte, red). **B**, Effect of the pre-incubation of the cells with 20 μM AEBSF on CO-induced changes in the rate of HEt fluorescence (representative trace from a single neuron, red). The lower traces in all panels of **A** and **B** (in black) depict the HEt signal after differentiation. The histograms below summarise the mean rates of cytosolic ROS production in the first 7 min (**C**) and 20 min (**D**) of CO exposure and after reoxygenation (**E**) of cortical neurons and astrocytes without and after pre-incubation with XO and NADPH oxidase inhibitors. Data shown as percentage from a basal rate of HEt fluorescence in control cells (100%) and are represented as mean ± SEM; ***p < 0.0001. (For interpretation of the references to colour in this figure legend, the reader is referred to the Web version of this article.)

The secondary phase of the activation of ROS production in response to CO exposure in neurons and astrocytes (Fig. 4A–D) was dependent on the presence of the xanthine oxidase inhibitors, Allopurinol (20 μM; N = 6 experiments) or Oxypurinol (20 μM; N = 4). It should be noted that the effect of CO in neurons was more pronounced than the effect in astrocytes. It should be noted that this secondary increase in ROS generation after CO application was similar to the effect of OGD deprivation [3], although activation of XO in CO-exposed neurons appeared earlier. Therefore, activation of ROS production in neurons and astrocytes at about 17–20 min after commencing CO exposure was induced by the activation of XO.

### 3.6 Post-CO activation of ROS production is induced by overactivation of NADPH oxidase

Washing CORM-401 out of the medium resulted in a profound acceleration of ROS generation in both astrocytes (5.2-fold to basal ROS level; Fig. 2A, D) and neurons (6.25-fold increase, Fig. 2 B, D). This increase in the production of ROS was affected by the presence of AEBSF, or DPI; inhibitors of NADPH oxidase (Fig. 4 B, E). DPI is also known to have an effect on mitochondrial complex I, but in low concentrations (0.5 μM, 20 min pre-incubation) DPI did not change mitochondrial potential but successfully inhibited NADPH oxidase [2,3]. Although post-CO activation of ROS production was much higher in neurons, both inhibitors successfully and almost completely inhibited the excessive increase in HEt fluorescence. It should be noted that the inhibition of NADPH oxidase in neurons and astrocytes had no effect on the first two phases of ROS production during CO exposure (Fig. 4 A, B). Thus, activation of ROS production in the post-CO exposure period is due to the activation of NADPH oxidase in neurons and astrocytes.

### 3.7 CO-induced lipid peroxidation in neurons and astrocytes

**Fig. 5:**
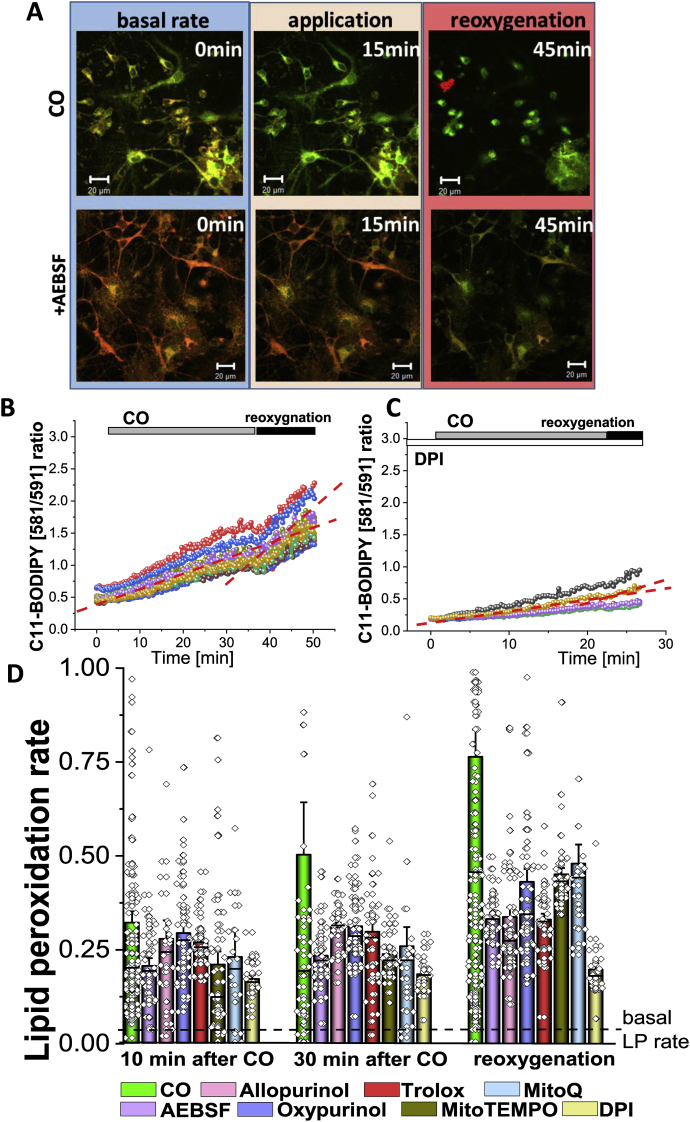
**Exposure of mixed primary cultures to CO increases lipid peroxidation.** **A**, Representative images of primary co-cultures loaded with C11-Bodipy in the presence of CO (upper panels) and when pre-incubated with AEBSF (lower panels). Scale = 20 μm. Representative traces of lipid peroxidation in primary neurons upon application of 60 μM CORM-401 (**C**) and when pre-incubated with DPI (0.5 μM, **B**). **D**, Quantification of lipid peroxidation rate in primary neurons and astrocytes pre-incubated with various enzyme inhibitors after application of CO at 10, 30 min and after reoxygenation. Bar indicates the incubation period of oligomers. n = 3 experiments. Error bars indicate SEM.

We have studied whether CO-induced production of ROS from mitochondria, XO, and NADPH oxidase in neurons and astrocytes could further activate lipid peroxidation. Using BODIPY C-11 as a fluorescent indicator for lipid peroxidation, we have found that application of CORM-401, but not of iCORM-401, produced an elevation in BODIPY C-11 ratio in the first 5–10 min (Fig. 5 A, B, D). It should be noted that CO exposure also produced a secondary XO-mediated increase in lipid peroxidation which was observed in experiments while measuring ROS using HEt (Fig. 2, Fig. 4). However, the most pronounced activation of lipid peroxidation in neurons and astrocytes was at the time of re-oxygenation, i.e. after washing the CORM-401 out of the medium (Fig. 5A and B,D). Importantly, the activation of lipid peroxidation could be blocked by mitochondrial antioxidants (Fig. 5 D) and XO inhibitors, and in the post-CO (re-oxygenation) phase, activation of lipid peroxidation could be blocked by inhibitors of the NADPH oxidase (Fig. 5C, D). Thus, the rate of lipid peroxidation was increased mainly due to the ROS production in mitochondria, and due to the NADPH oxidase in the reoxygenation period.

### 3.8 CO-mediated decrease of GSH

**Fig. 6:**
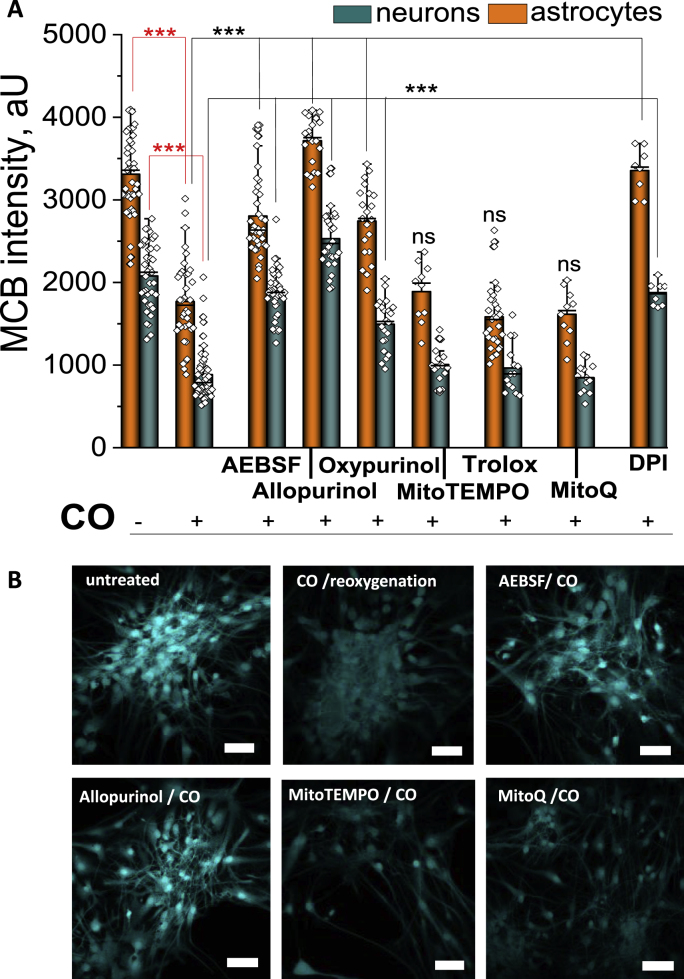
**Exposure of neuronal cultures to CO reduces endogenous GSH levels.** **A**, Quantification bar chart of the treatment of neurons (teal) and astrocytes (orange) with various inhibitors, preventing the decrease in endogenous GSH levels caused by CO-induced ROS production. **B**, Representative images of reduced GSH levels of rat primary cultures as assayed using MCB when untreated, after incubation with CO for 30 min, and pre-incubated with various inhibitors; Scale bar = 200 μm, Data are represented as mean ± SEM. ***p < 0.001, n.s. non-significant (red stars–decrease, black stars-decrease). (For interpretation of the references to colour in this figure legend, the reader is referred to the Web version of this article.)

Co-cultures of cortical neurons and astrocytes were treated for 40 min with CORM-401 followed by a removal of CORM-401 from the medium for 30 min. After loading the cells with 50 μM MCB, levels of glutathione were measured. A 40 min duration of CO exposure resulted in a significant decrease in GSH levels in both neurons and astrocytes compared to the untreated control (Fig. 6A and B). Importantly, pre-incubation of the cells with a water-soluble vitamin E analogue, Trolox (20 min, 100 μM Trolox), or with mitochondria-targeting antioxidants, MitoQ (100 nM), or MitoTEMPO (100 nM) did not change the effect of CO on the level of GSH in neurons or astrocytes (Fig. 6A and B). This suggests CO-induced mitochondrial ROS has a minor effect on the oxidative status of both types of cells. Furthermore, one might reason that the use of non-specific antioxidants is not protective against CO-induced depletion of cellular glutathione. However, XO inhibition had supressing effects on the CO-induced activation of lipid peroxidation (*see* Fig. 5D). This further resulted in the two inhibitors of XO (Oxypurinol and Allopurinol), reducing the effect of CO on GSH levels in neurons and astrocytes (Fig. 6A and B). However, the most effective compounds against CO-induced decrease of intracellular GSH levels were the inhibitors of NADPH oxidase, AEBSF (20 μM) and DPI (0.5 μM; Fig. 6A and B). Thus, CO induces depletion of the GSH pool in neurons and astrocytes due to an enzymatic overproduction of cytosolic ROS in NADPH oxidase and XO.

### 3.9 Effect of CO on neuronal and astrocytic viability; role of ROS

**Fig. 7:**
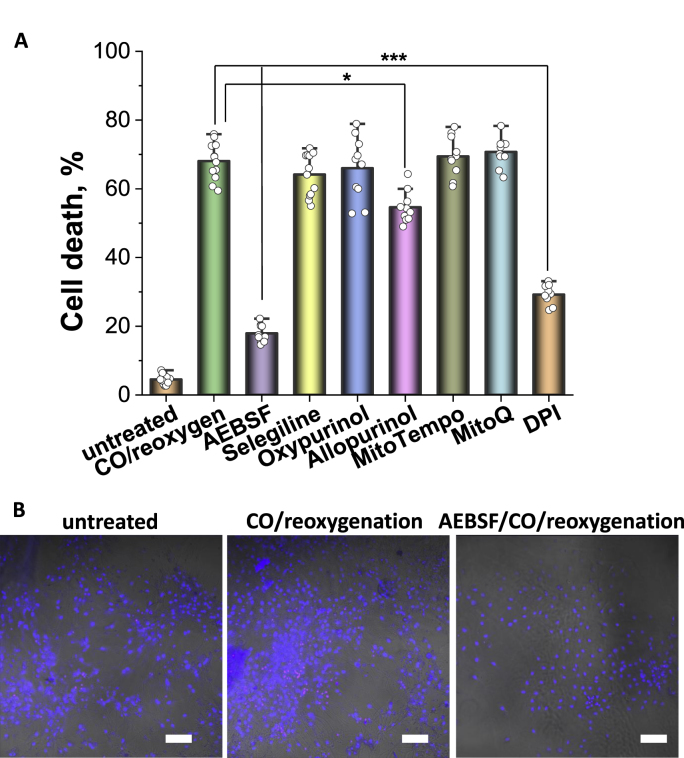
**CO-induced neurotoxicity can be prevented by inhibition of NADPH oxidase. A**, Cell death rate assessed in primary co-culture of neurons and astrocytes upon the application of 60 μM CORM-401 and after pre-treatment with various enzyme inhibitors **B**, Representative images of a cell death imaging essay in untreated, primary culture upon application of CO and reoxygenation and CO/reoxygenation effect in the presence of NADPH oxidase inhibitor AEBSF, 20 μM. Red: propidium iodide, non-viable cells; Blue: Hoechst, total number of cells, both merged with a bright field image. Scale bar = 200 μm. Data are represented as mean ± SEM. ∗p < 0.05, ∗∗∗p < 0.000. (For interpretation of the references to colour in this figure legend, the reader is referred to the Web version of this article.)

40 min incubation of the primary culture with CORM-401, followed by a 30 min-washout and a 24 h-re-oxygenation, caused the death of 67.70 ± 3.98% of the cortical neurons, and 54.2 ± 6.8% of the astrocytes from a primary co-culture (N = 4 experiments, Fig. 7). Pre-treatment of the cells with MitoQ (100 nM) or MitoTEMPO (100 nM) to reduce mitochondrial ROS generation did not protect neither neurons nor astrocytes against cell death (58.4 ± 6.4% (MitoQ) and 56.8 ± 5.1% (MitoTEMPO), nor cortical neurons or astrocytes 70.1 ± 4.1% (MitoQ) and 69.5 ± 5.5% (MitoTEMPO), respectively N = 5 experiments, Fig. 7).

Inhibition of XO with 20 μM Allopurinol, but not with 20 μM Oxypurinol (n.s.), was partially protective by reducing cell death in cortical neurons to 54.7 ± 4.5%, and in astrocytes to 36.8 ± 5.1%, (N = 5 experiments, Fig. 7).

Inhibition of enzymatic ROS production in NADPH oxidase with AEBSF (20 μM) or DPI (0.5 μM) dramatically reduced cell death to 17.4 ± 3.1% and 28.9 ± 4.5%, respectively for cortical neurons and to 19.2 ± 1.7% and 13.5 ± 2.2% respectively for astrocytes, (N = 6 experiments, both (p < 0.0001), Fig. 7).

## 4 Discussion

The extent of the known mechanisms of CO exposure that result in hypoxic injury of the tissues is well documented; with oxygen therapy as the treatment administered for attaining the best possible outcome for the patient. A complete mechanistic explanation of the delayed neurological sequelae that can persist following CO poisoning has been harder to provide and highlights gaps in knowledge and evidence base. However, it is widely accepted as occurring as a consequence of reperfusion injury, and more generally, the production of ROS causing neuronal cell death.

From this study, it has become clear that CO neurotoxicity is induced by ROS overproduction from the combination of at least 3 different ROS sources, followed by oxidative stress. CO poisoning and specifically neurotoxicity were previously suggested to be associated with oxidative stress and free radicals [31]. Many studies have suggested general antioxidant therapy as a protective therapy, although this has been largely unsuccessful. In this study, the effect of CO on ROS production was in the majority of the tests undertaken, similar to the effects seen in other research; due to chemical hypoxia or oxygen glucose deprivation [3], (Fig. 1, Fig. 2), although a more pronounced effect was seen in NADPH oxidase in the post-CO phase (re-oxygenation).

One of the most important findings of this work is that the majority of the oxidative damage that leads to brain cell death is induced by an enzymatic overproduction of ROS in NADPH oxidase during the phase of oxygen re-introduction, following CO exposure. This could provide an explanation for the negative neurological effects and the development of DNS in patients following CO poisoning, when 100% hyperbaric, or normobaric oxygen supplementation is used as a first line of treatment [14,16,30]. Activation of NADPH oxidase in neurons and astrocytes in the post-CO re-oxygenation phase is more likely stimulated by a mechanism similar to hypoxia or anoxia, by the activation of the calcium signal in these cells through glutamatergic or purinergic systems [2,3,18]; [2,39]. An increase in the levels of expression in several NOX enzymes was shown in the rat striatum after CO poisoning. This was associated with higher rates of hydroxyl radical production [17]. Importantly, the effect of this overproduction of ROS following CO exposure could be prolonged, and we observed activated lipid peroxidation for more than 1 h after washing CORM-401 out of the medium (Fig. 5). Inhibitors of NADPH oxidases have the potential to play neuro- and glia-protective roles against CO neurotoxicity for two major reasons – 1), most of the oxidative stress and cell death are induced by the overproduction of ROS from this enzyme and 2) because these inhibitors could be potentially used following CO poisoning, but prior to receiving 100% hyperbaric, or normobaric oxygen treatment. This is in contrast to other enzyme inhibitors, which predominantly work during CO exposure.

It should be noted that despite the difference in the isoforms of NOX in neurons (mostly NOX4) and astrocytes (mostly NOX2), both types of cells showed a massive increase in ROS in the post CO period and had an effect on GSH and lipid peroxidation independently of the type of ROS produced from the enzymes (hydrogen peroxide of superoxide).

One of the sources of ROS production and the consequent oxidative stress was XO, which had been previously shown to be a key component in the generation of ROS after CO poisoning [32]. Importantly, the inhibitor of XO, Allopurinol, effectively reduced neuronal death in the cerebral cortex and hippocampus and ameliorated cognitive deficits in rats after CO exposure [13]. Activation of the XO is more likely to be caused by a depletion of intracellular ATP, followed by conversion of adenine nucleotides to hypoxanthine and xanthine, which are substrates for XO [37]) [3,24,25]. This has also been confirmed by our results which indicate energy deprivation at the time of XO activation (15–25 min, Fig. 1). Interestingly, the production of ROS by XO did not induce a second phase of activation of lipid peroxidation (Fig. 5), but inhibitors of XO significantly decreased the effect of CO on the GSH level (Fig. 6). This suggests that superoxide and hydrogen peroxide, which are produced by this enzyme, more readily and effectively oxidise proteins from the bulk aqueous phase of the cytoplasm, rather than lipids from the membranes. However, a CO-induced production of ROS by XO had a large and unexpected impact in neuronal and astrocytic cells.

Mitochondria produce ROS in response to inhibition of the electron transport chain by CO [4]. Importantly, we found that mitochondria in astrocytes produced ROS much more intensively than neurons, although CO induced similar levels of mitochondrial depolarisation in both these types of cells (Fig. 1). Inhibition of mitochondria by CO is the initial step of the toxic effects of CO, which includes massive ROS production and inhibition of ATP production in oxidative phosphorylation. In considering this, mitochondria attracted the attention of the researchers as a potential target for development of a therapeutic strategy for protection against CO neurotoxicity [35,40]. However, in our experiments mitochondrial ROS production had an effect on lipid peroxidation only in the first 10 min of CO exposure, and mitochondrial antioxidants were not protective against CO-induced cell death (Fig. 7).

Under physiological conditions, CO is produced by heme oxygenase and plays a regulatory function in different tissues. Even at relatively physiological concentrations, CO effects mitochondrial function and glucose metabolism; effects that may be enhanced with toxic concentrations of CO [19,22,23].

Thus, our results suggest that the neurotoxic effect of CO is due to a multiphasic overproduction of ROS in neurons and astrocytes. The kinetics around the activation of ROS production at the time of CO exposure, suggests that using mitochondrial antioxidants or XO, will only achieve neuroprotection if these compounds are used prior to CO exposure, whereas the inhibition of NADPH oxidase could be neuroprotective even at the time of re-oxygenation. This finding makes the protection of neuronal health using antioxidants that inhibit NADPH oxidase, a potential addition to the current practice of treating CO poisoning with oxygen therapy across paramedic practice, the hospital Emergency Department, and in hospital HBOT facilities. However, until such treatment can be used in practice, oxygen therapy should still be considered the most effective treatment in cases of CO poisoning, but importantly, alongside appropriate neurological follow-up and treatment. The identification of mechanisms in this study associated with CO poisoning and DNS, highlights the importance of awareness amongst healthcare professionals of the potential for DNS to occur in their patients, and to recognise that symptoms, particularly in cases of severe poisoning, are unlikely to resolve in the absence of exposure to CO alone. The requirement, therefore, to actively facilitate long term specialist neurological support in patients with moderate to severe cases of CO poisoning following oxygen therapy is encouraged, in the current absence of a neuroprotective therapeutic.
